# A Third Dose of SARS-CoV-2 mRNA Vaccine Improves Immune Response in Chronic Kidney Disease Patients

**DOI:** 10.3390/vaccines11051012

**Published:** 2023-05-22

**Authors:** Maria Cecilia Poli, Cecilia Vial, Emma Rey-Jurado, Natalia González, Lina Jimena Cortés, Juan Hormazabal, Carolina Ramírez-Riffo, Javiera de la Cruz, Camilo Ulloa

**Affiliations:** 1 Departamento de Pediatría, Clínica Alemana de Santiago, Santiago 7650568, Chile; cpoli@udd.cl; 2 Programa de Inmunogenética e Inmunología Traslacional, Instituto de Ciencias e Innovación en Medicina, Facultad de Medicina, Clínica Alemana Universidad del Desarrollo, Santiago 7610658, Chile; emmarey@udd.cl (E.R.-J.); javieradelacruz@udd.cl (J.d.l.C.); 3 Programa Hantavirus y Zoonosis, Instituto de Ciencias e Innovación en Medicina, Facultad de Medicina, Clínica Alemana Universidad del Desarrollo, Santiago 7610658, Chile; mcvial@udd.cl (C.V.); linacortes@udd.cl (L.J.C.); jhormazabal@udd.cl (J.H.); carolinaramirez@udd.cl (C.R.-R.); 4 Departamento de Medicina Interna, Unidad de Nefrología y Trasplante Renal, Clínica Alemana de Santiago, Santiago 7650568, Chile

**Keywords:** SARS-CoV-2, COVID-19, immune responses, vaccination, hemodialysis, kidney transplant, chronic kidney disease

## Abstract

Chronic kidney disease (CKD) patients have an increased risk of morbidity and mortality following SARS-CoV-2 infection. Vaccination in these patients is prioritized, and monitoring of the immune response is paramount to define further vaccination strategies. This prospective study included a cohort of 100 adult CKD patients: 48 with kidney transplant (KT) and 52 on hemodialysis without prior COVID-19. The patients were assessed for humoral and cellular immune responses after four months of an anti-SARS-CoV-2 primary two-dose vaccination scheme (CoronaVac or BNT162b2) and one month after a booster third dose of BNT162b2 vaccine. We identified poor cellular and humoral immune responses in the CKD patients after a primary vaccination scheme, and these responses were improved by a booster. Robust polyfunctional CD4^+^ T cell responses were observed in the KT patients after a booster, and this could be attributed to a higher proportion of the patients having been vaccinated with homologous BNT162b2 schemes. However, even after the booster, the KT patients exhibited lower neutralizing antibodies, attributable to specific immunosuppressive treatments. Four patients suffered severe COVID-19 despite three-dose vaccination, and all had low polyfunctional T-cell responses, underscoring the importance of this functional subset in viral protection. In conclusion, a booster dose of SARS-CoV-2 mRNA vaccine in CKD patients improves the impaired humoral and cellular immune responses observed after a primary vaccination scheme.

## 1. Introduction

In the absence of vaccination, patients with chronic kidney disease (CKD) are at high risk of morbidity and mortality due to SARS-CoV-2 infection [1]. This higher risk is explained by the impaired immunity associated with their primary disease and to the immunosuppressive drugs they receive [2]. Before vaccination in Chile, kidney transplantation (KT) patients had a 1.2- and 5.1-fold increased risk of SARS-CoV-2 infection and mortality, respectively, compared to the general population [3]. Overall, COVID-19 mortality was 15.4% in KT patients, 30% in hospitalized patients, and 50% in patients requiring invasive mechanical ventilation [4]. According to international guidelines, COVID-19 vaccination is recommended for this high-risk population. Since the beginning of the pandemic, several vaccines have been approved for this use. In Chile, after their urgent approval, a massive, risk-stratified vaccination began with two doses of CoronaVac or BNT162b2 as the primary scheme of vaccination in February 2021 [5]. The primary two-dose scheme showed an efficacy of 18% in preventing COVID-19 and 66% of its associated deaths in a Chilean hemodialysis cohort in contrast to an efficacy of 66% and death prevention of 86% in the general population [5,6]. Moreover, recent studies suggest a decay in antibody response in CKD patients [7], which prompted a recommendation from the Chilean Society of Nephrology, the Transplant Society, and the Ministry of Health to administer a third booster dose of BNT162b2 to CKD patients beginning in August 2021 [3].

Recently, several studies have explored the immune response after two-dose vaccination schemes in CKD patients. In general, low humoral responses were found in this vulnerable population compared to healthy individuals, especially in KT patients [8,9,10,11,12,13,14,15,16]. Cellular immune response after vaccination in this population is relevant given that antibody response tends to be deficient; however, only a few studies have assessed cellular immune response in this population, showing overall weak cellular immune responses in KT but not in hemodialysis (HD) patients after a primary two-dose mRNA vaccination [17,18,19,20,21,22]. Importantly, a third dose of mRNA or adenoviral vector vaccine improves the immune response in CKD patients [23,24].

Anti-SARS-CoV-2 immune response induced by vaccination has not been compared in both transplanted and hemodialysis patients receiving a primary scheme of inactivated virus vaccine and an mRNA booster as it occurred in Chile. Evaluating this response is relevant for making public health decisions in terms of vaccination strategies in this immunocompromised population. We determined cellular and humoral immune responses after vaccination schemes that consisted of a primary vaccination of mRNA (BNT162b2) or inactivated virus (CoronaVac) vaccine and a homologous or heterologous boost with mRNA vaccine in 100 CKD patients, including HD and KT patients.

## 2. Materials and Methods

### 2.1. Study Protocol and Participants

This was a multicenter, prospective study of 100 adult patients with chronic kidney disease (CKD) who were undergoing hemodialysis or had a kidney transplant. All the patients enrolled had already received two doses of SARS-CoV-2 vaccine (CoronaVac or BNT162b2). All the participants received a BNT162b2 booster dose five months after the primary scheme. The exclusion criteria were the following: patients who had prior COVID-19, patients with a kidney transplant of less than 1 month or with active glomerular disease undergoing immunosuppressive treatment, and patients younger than 18 years of age. A group of 15 healthy individuals vaccinated with two doses of CoronaVac were included as controls, and all the participants received a BNT162b2 booster dose. Blood samples were taken at the first visit (120 days after the two-dose primary schedule (BNT162b2 or CoronaVac)) and second visit (30 days after the third, booster dose of BNT162b2). The patients were followed for five months after the last sample to report SARS-CoV-2 infection. The study was conducted in accordance with the Declaration of Helsinki. It was approved by the Clínica Alemana Universidad del Desarrollo Research Ethics committee, number 1049. All the patients signed informed consent except for the anonymized healthy controls, for whom the Clínica Alemana Universidad del Desarrollo Research Ethics committee granted a written exemption of this requirement.

### 2.2. Cellular Immune Response

Peripheral blood mononuclear cells (PBMCs) were isolated from peripheral blood using Histopaque-1077 (Sigma, St. Louis, MO, USA) density gradient as previously described [25], and stored frozen in liquid nitrogen in fetal bovine serum 10% dimethyl sulfoxide until use. The PBMCs from the HD and KT patients and the controls were thawed and counted. The assays could not be performed for samples with a low cell count. The PBMCs were stimulated for 24 h with a pool of native SARS-CoV-2 peptides (Peptivator Miltenyibiotec) at 37 °C with 5% CO_2_, and ELISpot and intracellular flow cytometry assays were performed. Phorbol 12-myristate 13-acetate (PMA)/ionomycin (Sigma, St. Louis, MO, USA) and cytomegalovirus peptides (Peptivator^®^, Miltenyi Biotec, Auburn, CA, USA) were added as positive controls and unstimulated as a negative control as previously described [26]. For Enzyme-linked Immunospot (ELISpot), human IFN-γ single-color ELISpot (CTL, Immunospot^®^, Shaker Heights, OH, USA) was performed according to the manufacturer’s procedures. Counting of spots was performed using ImageJ software. Flow cytometry staining was performed in individuals where an appropriate quantity of PBMCs was available. For intracellular flow cytometry staining, stimulated PBMCs were treated with Brefeldin A (Biolegend, San Diego, CA, USA) and GolgiStop (BD Biosciences, San Jose, CA, USA) and incubated for five hours at 37 °C with 5% CO_2_. After incubation, the cells were stained with viability-staining Near-IR (ThermoFisher, Waltham, MA, USA), permeabilized, and stained with surface and intracellular cytokine markers: CD3-V500 Clone UCHT1 (BD Biosciences, San Jose, CA, USA), CD4-FITC Clone A16141 (BD Biosciences, San Jose, CA, USA), CD8a-BV785 Clone RPA-T8 (Biolegend, San Diego, CA, USA), IFN-γ-AF700 Clone B27 (Biolegend, San Diego, CA, USA), IL-2-APC MQ1-17H12 (BD Biosciences, San Jose, CA, USA), CD25-PE-Cy5 BC96 (BD Biosciences, San Jose, CA, USA), TNF-α-PE-eFluor Clone MAb11 (ThermoFisher, Waltham, MA, USA), CD45RA-PE-Cy7 Clone HI100 (BD Biosciences, San Jose, CA, USA), and CCR7-PB Clone GO43H7 (Biolegend, San Diego, CA, USA). The cells were fixed and acquired in a Cytoflex LX cytometer (Beckman Coulter, Brea, CA, USA) and then analyzed using FlowJo software v.9.1. The proportions of cytokine-producing T cells in stimulated conditions were normalized to unstimulated cells. Positive cellular immune response was defined as more than 20 spots forming cells (SFC)/million cells in ELISpot and/or more than 0.01% of IFN-γ^+^ CD4^+^ or CD8^+^ T cells measured by flow cytometry. To evaluate polyfunctional CD4^+^ and CD8^+^ T cells, the Boolean gates strategy was performed using FlowJo software v.9.1. Visualization and statistics of proportion of the polyfunctional T cells were performed using SPICE v6.1 software [27]. To evaluate the memory T subsets cells, the effector memory T cells (TEM, CCR7^−^CD45RA^−^), central memory T cells (TCM, CCR7^+^CD45RA^−^), effector CD45RA^+^ T cells (TEMRA, CCR7^−^CD45RA^+^), and naïve T cells (TN, CCR7^+^CD45RA^+^) were gated.

### 2.3. Humoral Response

The enzyme-linked immunosorbent assay (ELISA) against SARS-CoV-2 Spike-IgG was performed to evaluate humoral response, as previously described [26]. Each sample was analyzed in duplicate, and the cutoff was set as the mean value of the negative controls (healthy donor pre-pandemic serum specimens) plus three standard deviations. Positive humoral response was defined as ELISA-positive samples. Neutralizing antibodies were measured using vesicular stomatitis virus (VSV)-green fluorescent protein (GFP)-Spike SARS-CoV-2, as previously described [26].

### 2.4. Statistical Analysis

GraphPad Prism v.9.1 software was used for comparisons of the immunological studies. The Fisher’s exact test was used to analyze contingency tables of categorical variables. The Mann–Whitney U test or the paired Wilcoxon signed-rank test were performed for comparison between the two unpaired or paired groups, respectively. The Kruskal-Wallis test followed by the Dunn’s multiple comparisons test was applied for comparisons between three or more groups.

## 3. Results

**Figure 1:**
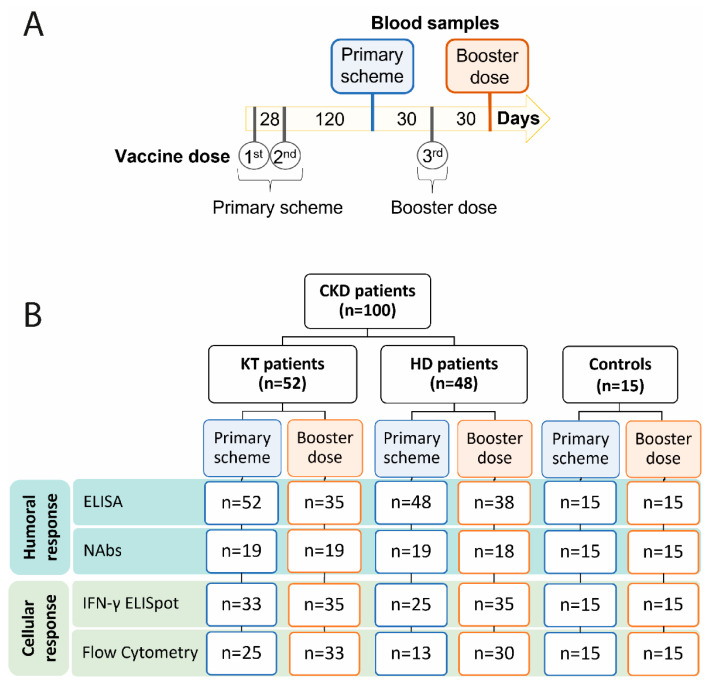
Study design. (**A**) Schematic representation of vaccine schemes and blood samples used in this study. (**B**) Number of assays performed for each group and time point. CKD: chronic kidney disease patients; HD: hemodialyzed patients; KT: kidney transplanted patients; Controls: healthy individuals; NAbs: neutralizing antibodies.

A total of one hundred CKD patients were recruited; 48 of these were in hemodialysis (HD) and 52 had received a kidney transplant (KT). The average age of our cohort was 68 and 54 in the HD and KT groups, with a Charlson comorbidity score of 6 ± 2.7 and 4 ± 2.1 points in the HD and KT patients, respectively. Specific renal diseases and immunosuppression schemes for both groups are described in Table 1. The patients were recruited after a primary vaccination scheme that included two doses of CoronaVac in 88% of the HD patients, while the other 12% received BNT162b2. In the KT group, 67% received a primary schedule with BNT162b2, while the rest received CoronaVac. The third vaccine dose was in all cases with BNT162b2, before and after which all the individuals were evaluated (Figure 1A). A group of fifteen healthy subjects was used as controls with an average age of 36 years and 67% female. They had a CoronaVac primary scheme and a BNT162b2 booster dose, and samples were taken at the same time points.

### 3.1. Humoral and Cellular Immune Response Increases Significantly after a Third Dose in CKD Patients

**Figure 2:**
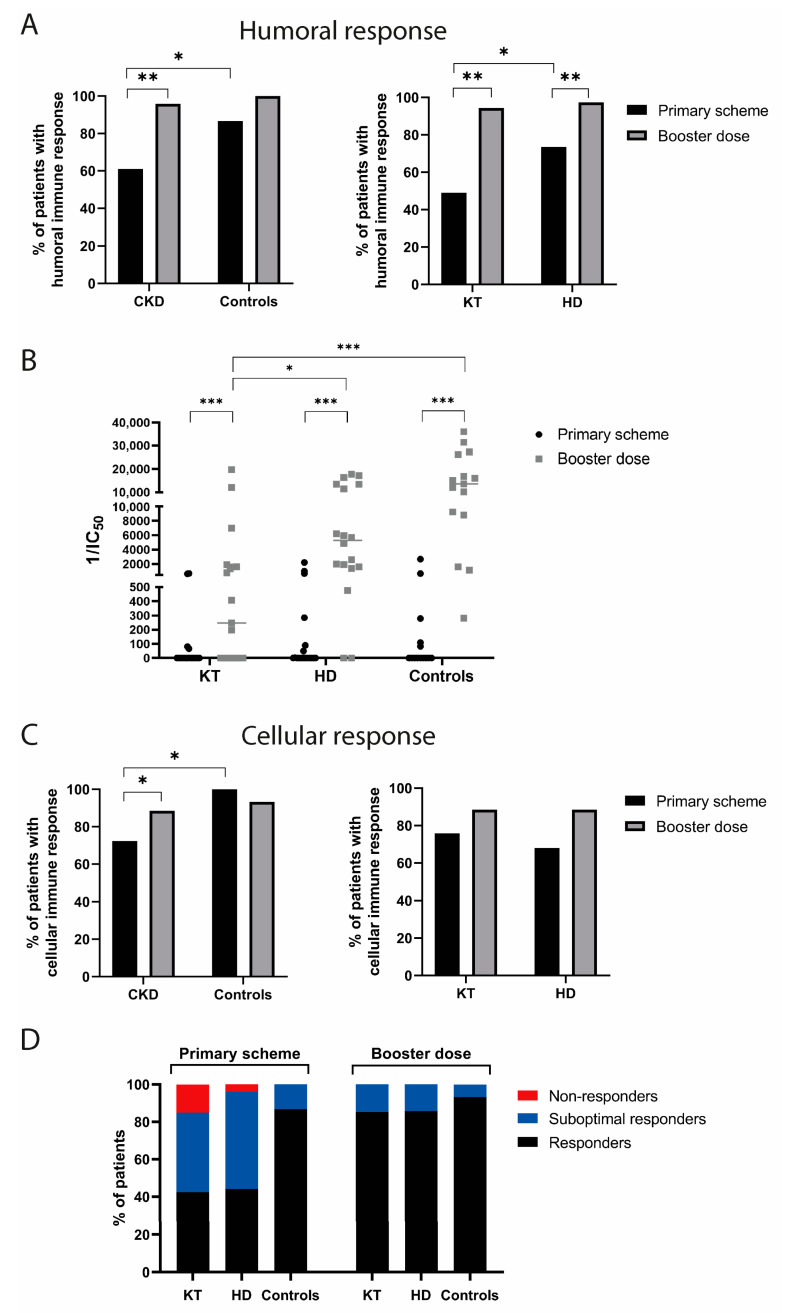
Increase in immune responders after the third dose in CKD patients. (**A**) Percentage of humoral responders after the primary scheme and a booster dose according to IgG-Spike ELISA. Fisher’s exact test (* *p* < 0.05; ** *p* < 0.01). (**B**) Neutralizing antibodies 1/IC_50_ comparison between groups. Paired Wilcoxon signed-rank test (* *p* < 0.05; ** *p* < 0.01; *** *p* < 0.001). (**C**) Percentage of cellular responders for each group and time point. Positivity according to IFN-γ ELISpot and IFN-γ expressing CD4^+^ or CD8^+^ T cells measured by flow cytometry. Fisher’s exact test (* *p* < 0.05). (**D**) percentage of non-responders, suboptimal responders, and responders according to IgG-Spike ELISA and IFN-γ ELISpot and flow cytometry. Categories of non-responders define patients without humoral and cellular immune response, “suboptimal responders” signifies patients having only cellular or humoral responses, and “responders” signifies patients who had both immune responses. CKD: chronic kidney disease patients; HD: hemodialyzed patients; KT: kidney transplanted patients; Controls: healthy individuals; IC_50_: half maximal inhibitory concentration.

Humoral and cellular immune response after vaccination is crucial to provide long-term protection for SARS-CoV-2 infection [28]. As immunocompromised patients may have a decreased capacity to mount such responses, it is crucial to determine both humoral and cellular immune responses with the various vaccine schemes available. The patients were first evaluated 4 months after a primary two-dose vaccine schedule and subsequently one month after a booster dose of BNT162b2 (Figure 1A). Humoral immune response was evaluated using ELISA to determine specific IgG against SARS-CoV-2 Spike protein and neutralizing antibodies, and flow cytometry and IFN-γ ELISpot assay were used to determine T cell–specific SARS-CoV-2 responses (Figure 1B). Humoral immune response was significantly lower in chronic kidney disease patients (61%) as compared to the controls (86%) after a two-dose primary vaccination scheme (Figure 2A). Comparison between the CKD groups showed that only 49% of the KT patients had a positive humoral immune response 4 months after the primary vaccine schedule, which was significantly lower compared to 73% of the HD group (Figure 2A). Importantly, 88% of the KT patients who had a positive humoral response after a two-dose primary schedule were vaccinated with BNT162b2 (Supplementary Figure S1), in line with the greater capacity of mRNA vaccines to induce stronger antibody responses, as previously reported [29].

The proportion of patients with a positive humoral immune response significantly increased in the CKD patients after a booster dose, similar to the healthy controls. Analyzing the CKD groups, the KT patients showed a larger increase (45%) compared to the HD (24%) after a booster dose (Figure 2A). Importantly, the levels of neutralizing antibodies significantly increased after the booster dose in all the groups. In contrast, significantly lower levels of neutralizing antibodies were found in the KT patients even after a third dose compared to the HD and control groups (Figure 2B).

We next sought to compare cellular immune responses using ELISpot and Flow cytometry. Positive IFN-γ T-cell responses after a primary scheme were also lower in the CKD patients compared to the controls (72% and 100%, respectively). After a third dose, cellular responses improved 16% in the CKD patients, reaching 88% of positivity (Figure 2C). Notably, 75% of the KT patients had positive cellular immune responses after a primary vaccination, despite only half of them having had positive humoral immune responses (Figure 2C), in line with a previous report [16].

In our cohort of CKD patients, we found no differences in the proportion of positive cellular or humoral responders comparing the CoronaVac and BNT162b2 schemes (Supplementary Figure S2), but the number of patients in the HD and KT groups with different schemes was too small to allow a separate analysis.

We defined patients who had neither humoral or cellular immune response as non-responders and patients who had only humoral or cellular responses as suboptimal responders. After the primary scheme, non-responders were only identified in the CKD group, and 1 and 5 patients were identified in the HD and KT groups, respectively. After a booster dose, all the patients were responders having either humoral, cellular, or both types of immune responses (Figure 2D). Altogether these results demonstrate that a booster dose is necessary to achieve a significant improvement in anti-SARS-CoV-2 immune response in CKD patients.

### 3.2. Booster Vaccination Elicits Robust Polyfunctional CD4^+^ T Cell Responses in CKD Patients

**Figure 3:**
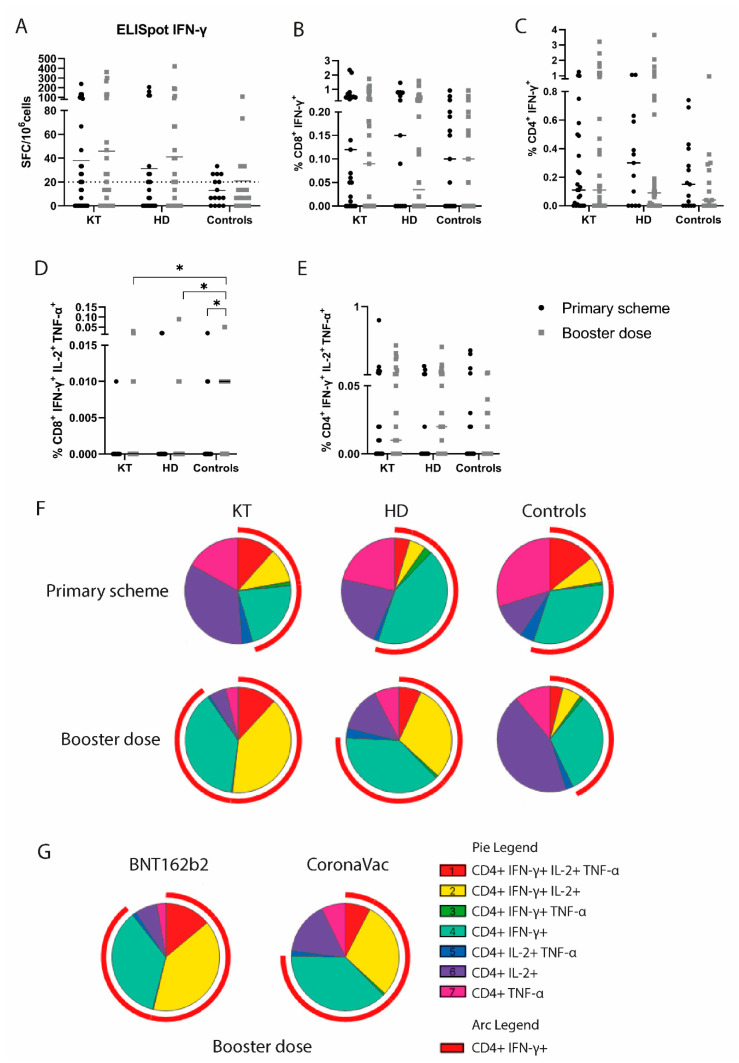
Monofunctional and polyfunctional T cell responses against SARS-CoV-2 after vaccination in chronic kidney disease patients. (**A**) Quantification of spot-forming cells (SFC) per million of cells by ELISpot assay after SARS-CoV-2 peptides stimulation. More than 20 SFC/106 was considered positive response. (**B**–**E**) Percentage of CD8^+^ IFN-γ^+^, CD4^+^ IFN-γ^+^, CD8^+^ IFN-γ^+^IL-2^+^TNF-α^+^, and CD4^+^ IFN-γ^+^IL-2^+^TNF-α^+^-producing T cells after primary scheme or booster dose measured by flow cytometry. Mann–Whitney test (* *p* < 0.05). (**F**) Proportion of monofunctional and polyfunctional cytokine–expressing CD4^+^ T cells for each group and time point. (**G**) Proportion of monofunctional and polyfunctional cytokine–expressing CD4^+^ T cells after a booster dose in chronic kidney disease patients. Patients were grouped according to primary scheme vaccines: CoronaVac and BNT162b2 (heterologous and homologous schemes, respectively). HD: hemodialyzed patients; KT: kidney transplanted patients; Controls: healthy individuals.

T cells mediate a specific SARS-CoV-2 cellular response by producing IL-2, TNF-α, and IFN-γ upon activation, and differential cytokine production by CD4^+^ and CD8^+^ T cells is important in protective antiviral responses [30]. T cell activation and cytokine production was evaluated upon exposure to SARS-CoV-2 peptide pools. All the groups showed no differences in IFN-γ spot-forming T cells after a booster dose measured by an ELISpot assay (Figure 3A). A similar trend was observed in the proportion of IFN-γ^+^-producing CD8^+^ and CD4^+^ T cells measured by flow cytometry (Figure 3B,C). Polyfunctional T cells producing two or more cytokines play a relevant role in viral immune response and provide long-term protection [31]. We sought to evaluate CD8^+^ and CD4^+^ polyfunctional T cells in vaccinated CKD patients. Even after a booster, the CKD patients had a decreased proportion of polyfunctional CD8^+^ T cells (CD8^+^IFN-γ^+^IL-2^+^TNF-α^+^) compared to the controls, suggesting persistently deficient immune responses in this group (Figure 3D). Additionally, the proportion of CD4^+^IFN-γ^+^IL-2^+^TNF-α^+^ tends to increase after a booster dose in HD and KT patients (Figure 3E), suggesting that a booster of mRNA vaccine is important to achieve CD4^+^ T cell polyfunctional responses in CKD patients. When analyzing cytokine-producing CD4^+^ T cells, we observed that after the primary scheme, the KT patients and the controls showed a higher proportion of triple positive CD4^+^ polyfunctional T cells (CD4^+^IFN-γ^+^IL-2^+^TNF-α^+^) than the HD patients (Figure 3F), consistent with a stronger T cell induction with mRNA vaccines in this group of immunocompromised patients [21,23]. After a booster dose, the KT patients showed a higher proportion of double (CD4^+^IFN-γ^+^IL-2^+^) and triple positive CD4^+^ T cells (CD4^+^IFN-γ^+^IL-2^+^TNF-α^+^) compared to both the HD and the controls (Figure 3F). Notably, 67% of the KT patients evaluated by flow cytometry received a primary scheme with two doses of BNT162b2, while 90% of the HD and 100% of the controls received two doses of CoronaVac, suggesting a stronger induction of polyfunctional CD4^+^ T cells with BNT162b2 vaccine. Moreover, the CKD patients who received three doses of BNT162b2 showed significantly greater proportions of polyfunctional CD4^+^ T cells as well as an overall proportion of IFN-γ-producing T cells than patients who received heterologous schemes (Figure 3G).

### 3.3. CD4^+^ and CD8^+^ IFN-γ Memory T Cell Response Increases in CKD Patients after a BNT162b2 Booster

**Figure 4:**
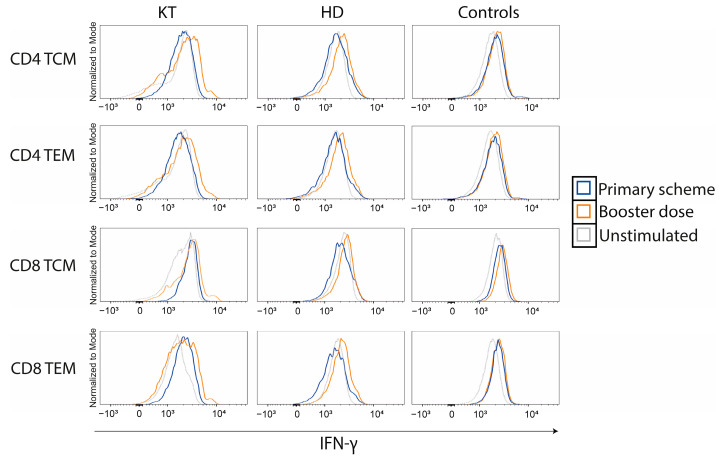
Memory T cell responses against SARS-CoV-2 increases after booster dose in chronic kidney disease patients. Histograms comparing IFN-γ^+^ responses in CD4^+^ and CD8^+^ T cell memory subsets TCM and TEM after primary scheme and after a booster dose. Unstimulated cells are depicted in dashed gray lines as a control. HD: hemodialyzed patients; KT: kidney transplanted patients; Controls: healthy individuals; TCM: central memory T cells; TEM: effector memory T cells.

Virus-specific memory T cells produce IFN-γ upon viral re-exposure and are crucial for providing long-time protection against SARS-CoV-2 [32]. To investigate this type of specific response in CKD patients, we evaluated the expression of IFN-γ in memory T cell subsets, both after the primary scheme and after a booster. Gating strategy to differentiate memory T cells is shown in Supplementary Figure S3. After a booster dose, we observed an increase in specific SARS-CoV-2 CD4^+^ and CD8^+^ IFN-γ^+^ responses in memory T cell subsets for all groups (Figure 4), highlighting the need of three doses to improve cellular immune responses in this at-risk population.

### 3.4. Immunosuppressive Drugs and Vaccine Response in KT Patients

**Figure 5:**
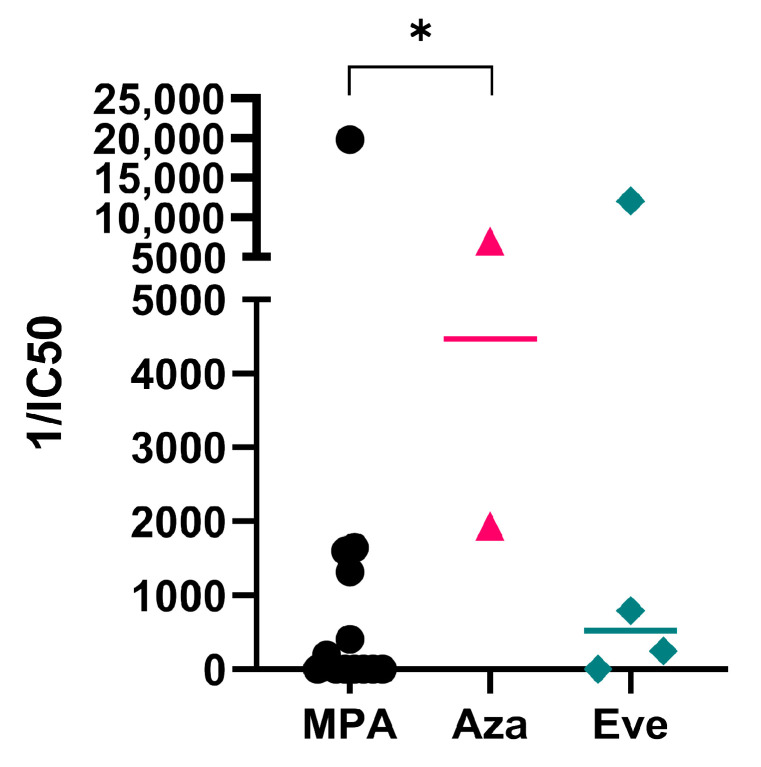
Lower neutralizing antibodies in mycophenolate-treated KT patients after a booster dose. Neutralizing antibodies 1/IC_50_ of kidney-transplanted patients after a booster dose. Patients were grouped according the third drug of tri-therapy of immunosuppressors that included tacrolimus, prednisone, and one of the following drugs: mycophenolic acid or sodium mycophenolate (MPA), azathioprine (Aza), or everolimus (Eve). Kruskal-Wallis test followed by the Dunn’s multiple comparisons test (* *p* < 0.05). HD: hemodialyzed patients; KT: kidney transplanted patients; Controls: healthy individuals; IC_50_: half-maximal inhibitory concentration.

All the KT patients were under immunosuppressive treatments including tacrolimus and prednisone as part of tri-therapy, and we hypothesized that this could influence protective immune responses. After a booster dose, no differences among different immunosuppressive therapies were observed in either the overall humoral or the cellular immune responses (data not shown). However, significantly decreased neutralizing antibody responses were found in patients who received mycophenolic acid or sodium mycophenolate (MPA) as part of immunosuppressive treatment (Figure 5). These results agree with previous reports showing lower humoral responses in mycophenolate-treated patients [33,34] and support a recommendation for additional booster doses in these individuals.

### 3.5. Outcome

**Figure 6:**
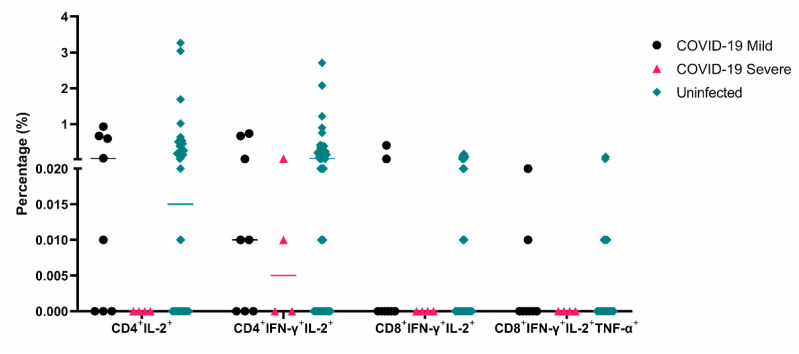
Lower monofunctional and polyfunctional T cell responses against SARS-CoV-2 in chronic kidney disease patients who had COVID-19 after vaccination. Percentage of CD4^+^ IL-2^+^ -, CD4^+^ IFN-γ^+^IL-2^+^ -, CD8^+^ IFN-γ^+^IL-2^+^ -, and CD8^+^ IFN-γ^+^IL-2^+^TNF-α^+^-producing T cells after a booster dose in patients who had mild (*n* = 8) and severe COVID-19 (*n* = 4) or who remained uninfected (*n* = 51) in the follow-up period.

After the enrolment phase of this study ended, three patients from our cohort died, two HD patients had sepsis, and one KT patient died of lung cancer (Table 1). Fifteen patients from our cohort had COVID-19 (8 KT and 7 HD patients), and four had severe disease and required hospitalization (2 KT and 2 HD patients). We found that the patients who had severe COVID-19 had low or absent cellular immune responses of CD4^+^IL-2^+^, CD4^+^IFN-γ^+^IL-2^+^, CD8^+^IFN-γ^+^IL-2^+^, and CD8^+^IFN-γ^+^IL-2^+^TNF-α^+^ after a booster dose (Figure 6), in line with the relevance of polyfunctional T cell responses against viruses [35].

## 4. Discussion

Impaired cellular and humoral immune responses after two-dose vaccination schemes are extensively reported in CKD patients, showing a stronger immune response in HD patients [9,17,18,21]. Most studies included mRNA vaccinated patients, and only a few studies evaluated the immune responses of CoronaVac schemes in a cohort that included KT patients [16,36,37,38,39,40] and/or HD patients [14,41,42,43,44]. Here we show that HD and KT patients have a reduced humoral and cellular immune response after a primary scheme with either CoronaVac or BNT162b2 that increases after a BNT162b2 booster.

Several studies have demonstrated SARS-CoV-2 vaccine effectiveness in the prevention of infection, severe illness, and COVID-19 hospitalization in immunocompromised groups [45]. Moreover, a single-center study in the US showed inferior survival of unvaccinated compared to vaccinated liver and kidney transplant recipients [46], reinforcing the importance of vaccination in these immunocompromised groups. Recently, an observational study in a Chilean cohort of HD patients showed a reduced risk of infection and longer survival of patients with a booster dose compared to single-dose and unvaccinated patients [47], consistent with the stronger humoral and cellular responses after a booster dose observed in our HD cohort.

We observed that the KT patients had an increased proportion of polyfunctional CD4^+^ T cells compared with the healthy donors, likely due to the different vaccination schemes used in these groups, given that most healthy individuals in Chile received only CoronaVac as a primary scheme. Consistently, we found increased proportions of polyfunctional CD4^+^ T cell responses in CKD patients receiving three doses of mRNA vaccine BNT162b2 compared to a CoronaVac primary schedule with a BNT162b2 booster. In contrast to our findings, Stumpf et al. showed no differences in the proportions of SARS-CoV-2-reactive polyfunctional CD4^+^ T cells between HD, KT, and healthy controls vaccinated with two doses of BNT162b2 or mRNA-1273 [13]. Another study showed that KT patients had significantly decreased frequencies of spike-specific IFN-γ^+^TNF-α^+^IL-2^+^ polyfunctional T cells compared to HD and healthy controls, and all of them had received two doses of BNT162b2 [17]. Comparison between vaccination schedules in a Chilean cohort of solid-organ transplant recipients, including KT patients, showed that homologous BNT162b2 scheme induced higher humoral responses but similar specific IFN-γ or IL-2 T cell responses compared to heterologous scheme [37]. Comparison of polyfunctional T cells between these two schemes has not been performed; therefore, our study is the first to show differences in a CKD cohort. Altogether these results suggest that differences in polyfunctional CD4^+^ T cells cannot be attributed only to CKD condition but also to other factors including immunosuppressive regimens and natural infection, among others. Nevertheless, heterologous regimens used in Chile reach polyfunctional CD4^+^ T cell responses comparable to homologous schemes used in this and previous studies.

Additionally, we found that triple polyfunctional CD8^+^ T cells remain significantly lower in CKD patients even after a booster vaccination. It has been shown that polyfunctional CD8^+^ T cells have enhanced effector function and correlate best with protection in viral infections such as HIV [48,49], and we hypothesize that they could play an important role in SARS-CoV-2 protection. Interestingly, we found that the patients in our cohort who suffered severe COVID-19 after vaccination had low or absent CD4^+^ and CD8^+^ mono- and poly-functional T cell responses after a booster dose, highlighting the importance of cellular responses to achieve protection against viral infections and supporting this hypothesis [31]. In this context, monitoring polyfunctional T cell responses could help to predict infection risk or booster requirement in CKD patients. However, due to the small sample size of our cohort, further studies are needed to confirm this hypothesis.

Similar to other reports with ChAdOx1 and mRNA vaccines [44,50], we observed that even after a third dose of BNT162b2 a proportion of KT patients do not have neutralizing antibodies, especially patients under mycophenolic acid or sodium mycophenolate treatments, consistent with previous reports showing decreased humoral responses in patients receiving mycophenolate [33,34,51]. It has been shown that mycophenolic acid significantly inhibits proliferation and differentiation of primary human B cells, impairing immunoglobulin secretion of activated but not terminally differentiated B cells [52]. A temporary mycophenolate hold occurring during the fourth dose administration showed augmented virus-neutralizing capacity and B cell responses in KT patients [53]. Comparable results were observed in the humoral responses of liver transplant recipients receiving a primary vaccination scheme during temporary suspension of mycophenolate [54]. These studies suggest that an adjustment of immunosuppressive treatments during booster vaccination could be a strategy to improve humoral responses in previously unresponsive patients.

Considering our findings, it is necessary to administer additional SARS-CoV-2 immunization in CKD patients to achieve optimal immune responses. Recent reports showed that a fourth booster dose of mRNA vaccine significantly increased anti-spike antibody titers and reduced COVID-19 breakthrough infections in HD and KT patients [55,56]. In Chile, a fourth dose was administered to the general population beginning in January 2022 with immunocompromised groups [57]; however, no efficacy studies are yet available, and further studies are needed to define vaccination strategies for this high-risk population.

The main strength of our study is the analysis of both humoral and cellular immune responses after the primary vaccination scheme and after a booster dose in a cohort that included HD and KT patients. Additionally, having a follow-up period of six months for COVID-19 breakthrough allowed us to identify immune markers related to outcome. A limitation of this study is the inclusion of an unequal number of KT and HD patients vaccinated with CoronaVac and BNT162b2, which restricts comparisons between different vaccination schemes separately in these groups. Another limitation is that all the healthy controls had received CoronaVac primary schemes, which prevented comparing results with BNT162b2 schemes. Finally, our study did not assess immunity against additional SARS-CoV-2 variants of concern, but other studies have shown that BNT162b2 vaccination induces CD4^+^ and CD8^+^ T cell responses that cross-recognize the Omicron variant in a healthy population [58].

In conclusion, two doses of vaccine are not enough to mount an effective cellular and humoral response in CKD patients. A third heterologous or homologous booster improves neutralizing antibodies titers and polyfunctional T cells in CKD patients as well as in healthy individuals. Even after three doses, some KT patients under immunosuppressive drugs show no neutralizing antibodies, suggesting the need of further vaccination in this population.

## Figures and Tables

**Table 1:** Cohort demographics.

Variables	Hemodialysis (*n* = 48)	Kidney Transplant (*n* = 52)
Age (mean years ± SD)	68.3 ± 13.9	53.7 ± 12.7
**Gender**		
Female	12 (25.0%)	17 (32.7%)
Male	36 (75.0%)	35 (67.3%)
BMI (mean kg/m^2^ ± SD)	26.2 ± 3.5	26.8 ± 4.6
**Primary vaccination schedule**		
CoronaVac	42 (87.5%)	35 (67.3%)
BNT162b2	6 (12.5%)	17 (32.7%)
**CKD etiology**		
Mellitus diabetes	15 (31.2%)	7 (13.5%)
Unknown	20 (41.7%)	13 (25.0%)
Glomerular	8 (16.7%)	18 (34.6%)
Congenital/genetic	4 (8.3%)	11 (21.1%)
Others	1 (2.1%)	3 (5.8%)
Time after kidney replacement therapies:Hemodialysis or transplant (mean days ± SD)	1915 ± 1786	2125.6 ± 6007.3
**Immunosuppression**	None of the patients received pharmacological immunosuppression.	
FK+MPA+PND		36 (69.2%)
FK+Aza+PND		4 (7.7%)
FK+Eve+PND		7 (13.5%)
FK+Rapa+PND		1 (1.9%)
CSA+Aza+PND		2 (3.8%)
Belatacept		2 (3.8%)
Charlson Score (mean ± SD)	6 ± 2.7	4 ± 2.1
Death	2 (4.2%)	1 (1.9%)

SD = standard deviation; BMI = body mass index; CKD = chronic kidney disease; FK = tacrolimus; MPA = mycophenolic acid or sodium mycophenolate; PND = prednisone; Aza = azathioprine, Eve = everolimus, Rapa = rapamycin; CSA = cyclosporine.

## Data Availability

All the patient data and the analysis are available online in Spreadsheet S1.

## Supplementary Materials

The following supporting information can be downloaded at: https://www.mdpi.com/article/10.3390/vaccines11051012/s1, Figure S1: ELISA against SARS-CoV-2 spike protein in KT patients; Figure S2: Positive immune responders with different primary vaccination schemes; Figure S3: Gating strategy for memory T cell populations; Spreadsheet S1: Data of patients.
