# Supplementary material for: A Third Dose of SARS-CoV-2 mRNA Vaccine Improves Immune Response in Chronic Kidney Disease Patients
Source: Vaccines (Basel). 2023 May 22;11(5):1012. doi: 10.3390/vaccines11051012 (PMC10221173; doi:10.3390/vaccines11051012)
Supplement: Supplementary file 1 [file vaccines-11-01012-s001.zip › supplementary figures.pdf]

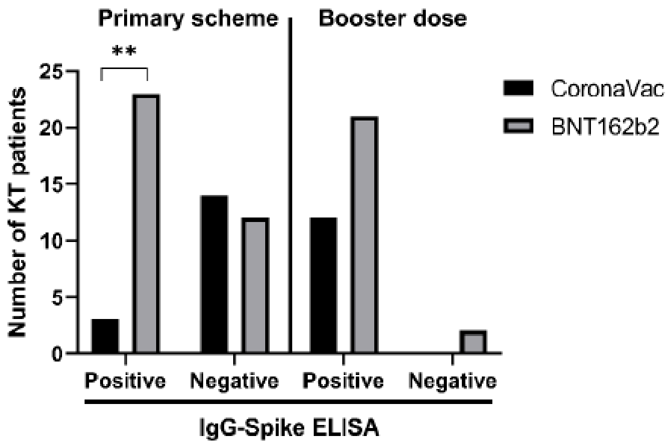

Figure S1. ELISA against SARS-CoV-2 spike protein in KT patients. Patients were grouped according to primary scheme vaccines (CoronaVac or BNT162b2). Graph shows the number of patients with positive or negative ELISA after primary scheme and after a booster dose. Fisher's exact test (** $p < 0.01$).

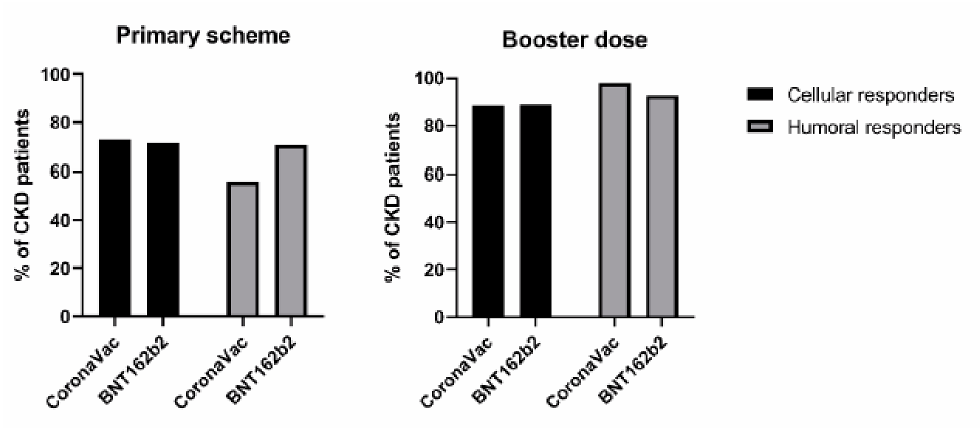

Figure S2. Positive immune responders with different primary vaccination schemes. CKD patients were grouped according to primary vaccination schemes (CoronaVac or BNT162b2). The graphs show the percentage of positive cellular or humoral immune responders in each group evaluated after the primary scheme and after a booster dose, as defined in materials and methods section.

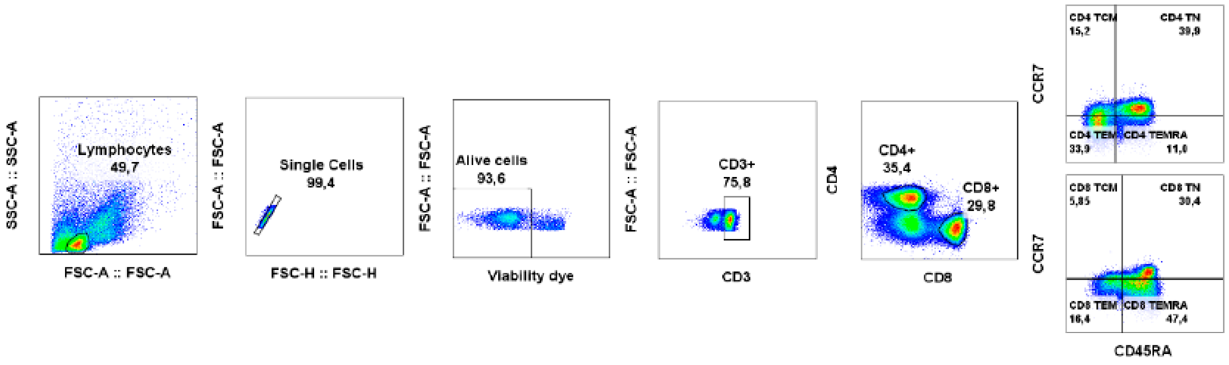

Figure S3. Gating strategy for memory T cell populations. CD4+ and CD8+ T cells were gated from live, singlets, CD3+ lymphocytes. Memory subpopulations were defined by expression of CCR7 and CD45RA. TEM: effector memory T cells (CCR7-CD45RA-), TCM: central memory T cells (CCR7+CD45RA-), TEMRA: effector CD45RA+ T cells (CCR7-CD45RA+) and TN: naïve T cells (CCR7+CD45RA+).
